# Amino acid function and docking site prediction through combining disease variants, structure alignments, sequence alignments, and molecular dynamics: a study of the HMG domain

**DOI:** 10.1186/1471-2105-13-S2-S3

**Published:** 2012-03-13

**Authors:** Jeremy W Prokop, Thomas C Leeper, Zhong-Hui Duan, Amy Milsted

**Affiliations:** 1 Department of Biology, Program in Integrated Bioscience, The University of Akron, Akron, OH USA; 2 Department of Chemistry, Program in Integrated Bioscience, The University of Akron, Akron, OH USA; 3 Department of Computer Science, Program in Integrated Bioscience, The University of Akron, Akron, OH USA

## Abstract

**Background:**

The DNA binding domain of HMG proteins is known to be important in many diseases, with the Sox sub-family of HMG proteins of particular significance. Numerous natural variants in HMG proteins are associated with disease phenotypes. Integrating these natural variants, molecular dynamic simulations of DNA interaction and sequence and structure alignments give detailed molecular knowledge of potential amino acid function such as DNA or protein interaction.

**Results:**

A total of 33 amino acids in HMG proteins are known to have natural variants in diseases. Eight of these amino acids are normally conserved in human HMG proteins and 27 are conserved in the human Sox sub-family. Among the six non-Sox conserved amino acids, amino acids 16 and 45 are likely targets for interaction with other proteins. Docking studies between the androgen receptor and Sry/Sox9 reveals a stable amino acid specific interaction involving several Sox conserved residues.

**Conclusion:**

The HMG box has structural conservation between the first two of the three helixes in the domain as well as some DNA contact points. Individual sub-groups of the HMG family have specificity in the location of the third helix, DNA specific contact points (such as amino acids 4 and 29), and conserved amino acids interacting with other proteins such as androgen receptor. Studies such as this help to distinguish individual members of a much larger family of proteins and can be applied to any protein family of interest.

## Background

Predicting function from protein sequence is a complex and challenging task. Multiple sequence alignments can give insights into functional conservation over evolutionary time but are limited to what can be observed at the level of primary structure. Combining these sequences with known protein tertiary structures provides a three dimensional explanation of potential evolutionary pressures, but correlating the conservation to specific functions is still a challenge. This study compares natural variants (NV) associated with disease phenotypes to molecular dynamic (MD) simulations of DNA binding, predicting the functionality of specific amino acids within a medically important protein domain.

The high mobility group (HMG) box is composed of three helices that make an "L" shape able to bind the minor groove of DNA (reviewed in [1]). Many of the members of this protein family bind to DNA with low sequence specificity, such as the HMGB1 protein important in inflammation response [2]. Some members, such as the Sox sub-family, bind to DNA with a higher degree of sequence specificity [3]. The Sox family consists of 20 known human proteins, with the most thoroughly studied being the mammalian testis-determining factor, Sry [4]. Recent work has shown Sry to have additional functions outside testis determination. These functions may include brain development [5,6], activation of the sympathetic nervous system [7], and blood pressure regulation [8]. Identifying and understanding the roles of conserved amino acids in Sry and other Sox proteins may lead to insights into particular amino acid functions. These might be HMG specific, such as DNA binding and structure, or specific to individual protein members. Combined analysis of amino acids known to have natural variants in disease phenotypes via multiple sequence alignment, structure alignment and MD simulation reveals several amino acids in the Sox family that may contribute to Sox specific functions such as interactions with the androgen receptor (AR).

## Methods

### Natural variants

Natural variations of amino acids in HMG proteins associated with various diseases were collected from Uniprot [9] and can be seen in the Additional file 1 along with all sequence accession codes. These amino acids were highlighted on the sequence of Sry, which could be used to identify conserved regions on multiple sequence alignments.

### Sequence and structure alignments

All sequence alignments were performed with ClustalW [10] using the BLOSUM62 matrix [11]. Human HMG proteins were retrieved from Uniprot, and proteins containing multiple HMG domains were parsed into individual domain sequences. Human sequences were used to study conservation of the HMG family, while sequences from multiple species (from invertebrates to vertebrates) were used in studying conservation of an individual member of the family across evolutionary time. HMG protein structures were identified by blasting the sequence of the HMG box of Sry against the Protein Data Bank (PDB) [12] using blastp from NCBI with default settings [13]. All structures were cleaned by removing all molecules (water, salts, DNA, additional protein sequence) that were not part of the HMG domain. For NMR structures containing multiple models, only regions of high agreement from the first reported ensemble member were used. The multiple structures of HMG proteins were superposed using MUSTANG [14] to the structure of SRY remaining bound to DNA. Sox proteins were also superposed to identify Sox-specific features.

### Molecular dynamic (MD) simulations

All MD simulations were run using YASARA Structure [15] with Amber03 force field [16] for 1000 picoseconds (ps). The md_run macro [17] was used with a water density of 0.997g/mL. Simulations were analyzed using both the md_analyse and md_analyzeres macros [17]. Structure 1j46 was used for MD of SRY. As no known structure exists for Sox9, models were created using I-TASSER [18], superposed onto DNA using structure 1j46, and the energy was minimized with YASARA. Although HMGB1 contains two HMG domains, only the second (which contains the NV) was used to run the MD simulation of HMGB1. Amino acid substitutions were performed by swapping amino acids in YASARA.

### Sry-AR predicted interactions

A short peptide of the AR was docked into the model by placing the fragment in close proximity to the proposed contact amino acids of SRY and the energy of the system was minimized *in vacuo*. The starting model for docking was derived from 1j46 coordinates. The model was placed in simulation space of 57, 72, and 57 Å, water was added to the system at 0.997g/mL, and the system was energy minimized. Three different simulations were run on both SRY and Sox9 for 1500 ps each: docked AR (Docked), AR in which all the amino acids were swapped with alanine (Docked A's) to show sequence specificity, and the AR pulled away from interaction (free). Movement of the AR peptide in each system was recorded over the simulation every 25 ps. Sox9-AR interactions were investigated by replacing amino acids in the structure of Sry with those present in Sox9.

## Results and discussion

**Figure 1:**
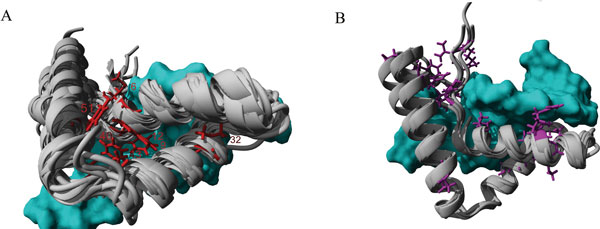
**A) Structure alignments of 18 HMG box domains from various proteins bound to DNA (cyan)**. Amino acids in red were NVs conserved 90% or greater in the 69 HMG sequences. B) Alignments of 7 SOX specific HMG box structures with NV amino acids in purple conserved 90% or greater in the 20 human SOX sequences.

Forty four natural variants (NVs) were found in 33 amino acids in a total of 5 HMG proteins. All NVs were found in Sox members except for two in the HMGB1 protein. When aligning the 18 known structures of HMG proteins, it appears that the first and second helixes have a high degree of structural conservation, while the third helix varies considerably (Figure 1A). The divergence in the geometry of this third helix is likely due to changes in kinetics and thermodynamics of protein folding and DNA binding between individual members. Forty nine non-Sox human HMG domain sequences were identified and 20 human SOX, totaling 69 human HMG protein sequences. Eight NV amino acids are conserved with 90% or greater frequency in these sequences. Four hydrophobic, aromatic amino acids (9, 12, 40, 51) were conserved between the first, second, and beginning of the third helix that likely contribute to proper packing of the three helices. Additionally a hydrophobic amino acid is conserved at amino acids 6, 32, and 43 contributing to structural organization and non-sequence specific DNA interaction, while a conserved polar basic amino acid is found at 48 associated with non-sequence specific DNA interaction.

Most NVs were conserved in Sox family members rather than in non-Sox HMG sequences. Because of the paucity of NVs in non-Sox HMG proteins, and with only 8 of the 33 NV amino acids conserved in the HMG family sequences, we decided to determine if any amino acids were conserved only in the Sox family. Nineteen additional amino acids with NVs were conserved 90% or greater in the Sox family with the previous 8 HMG NVs also conserved in SOX. Structure alignments of the Sox family members show a highly conserved first, second and third helix (Figure 1B) with several clumped regions of conserved NVs. A hydrophobic core is conserved between the N-terminus and the C-terminus of the Sox proteins. All of the NVs involved in Sry based disease associations were conserved in multiple sequence alignments of Sry.

**Figure 2:**
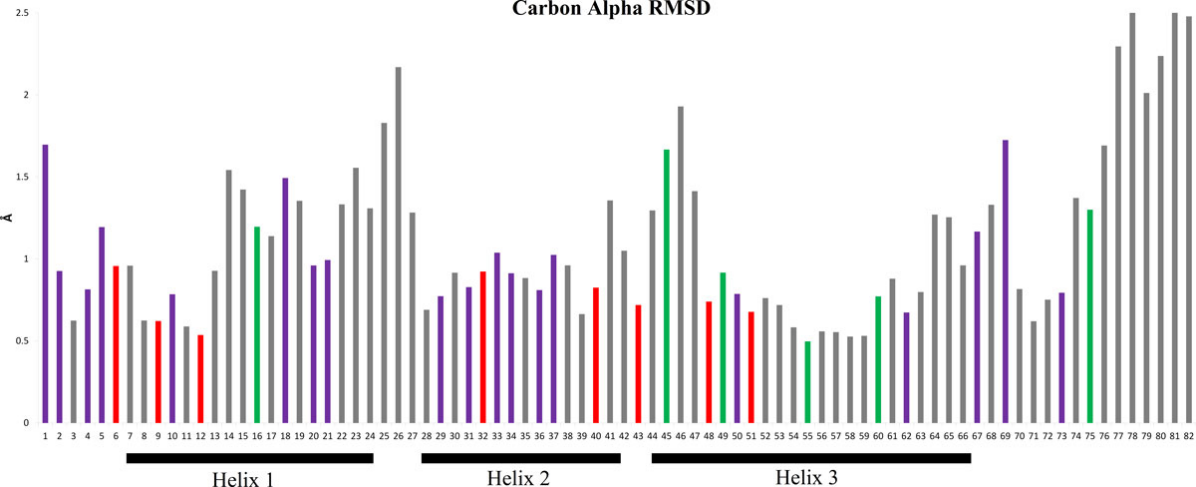
**MD simulations of SRY [PDB: **1j46**] showing the carbon alpha RMSD for each amino acid**. Colors of the bars represent NV and where they were conserved on sequence alignments: red = HMG, purple = SOX, green = not conserved, grey = no NV.

During MD simulations the movement of each amino acid can be tracked relative to the starting structure. Figure 2 shows the averaged root mean square distance (RMSD) of the alpha carbon for each amino acid from the initial structure to the structure every 25ps of MD simulation. It can be seen that the amino acids that are highly conserved in the HMG sequences (red Figure 2) have a lower RMSD (around 1 or below). Thus these amino acids deviated less during the simulation, suggesting that these amino acids are in low energy conformations with strong energetic costs associated with their structural perturbation. We ascribe this to their contribution to structural packing and/or DNA interaction. For example, some of the amino acids that are conserved in the Sox family and appear to contribute to DNA specificity in binding, such as amino acid 4 and 29, have lower dynamics during the simulation (purple Figure 2).

**Figure 3:**
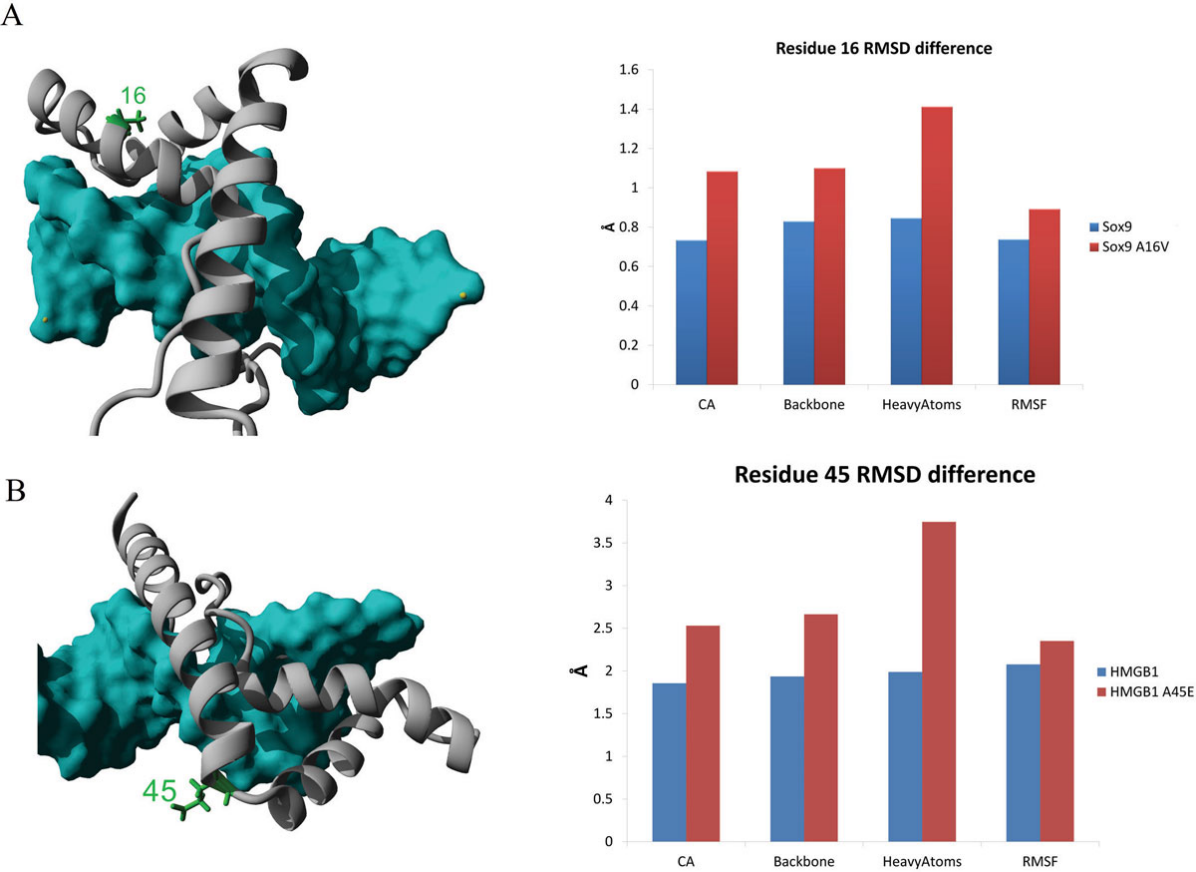
**A) NV at amino acid 16 (green) located on the back side of DNA binding on helix 1**. MD simulations show increased movement of the NV (red) compared to the normal Sox9 (blue) at amino acid 16. B) NV at amino acid 45 (green) at the beginning of the third helix. An acidic amino acid leads to an increase in movement (red) over the normal alanine (blue).

Six amino acids with disease associated NVs were not conserved in either the HMG or Sox family, two of which are of particular medical interest. Amino acid 16 is found as a valine in place of alanine in a Campomelic dysplasia patient, but this substitution showed little effect on DNA binding [19]. Models of Sox9 structure show the amino acid to be on the opposite side of the HMG box DNA binding (Figure 3A). Of the 69 HMG sequences only SOX8, SOX9, and SOX10 had an alanine. Sixty of the 61 SOX9 sequences from multiple species, 16 out of 16 Sox8, and 12 out of 13 Sox10 had a conserved alanine at this amino acid suggesting a possible functional conservation found only in these proteins. MD simulations of the NV show an increase movement of the amino acid (Figure 3A), which we propose changes the kinetics or thermodynamics of interaction with another protein most likely involved in nuclear localization. Amino acid 45 is found to have a substitution of a glutamic acid from an alanine in a gastric carcinoma cell line HMGB1 protein [20]. In simulations this amino acid is highly dynamic and does not appear to contribute to DNA interaction (Figure 3B), but may contribute to interaction with another protein.

**Figure 4:**
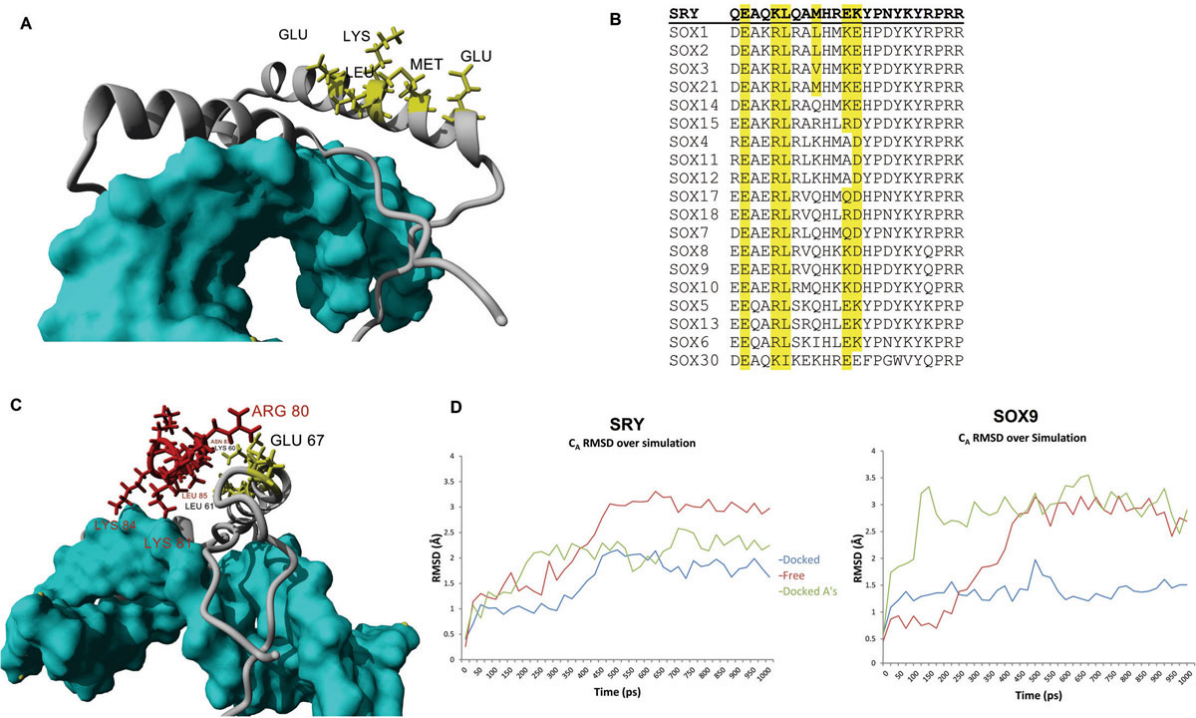
**A) Amino acids (yellow) located on the backside of the third helix conserved in multiple sequences of Sry not identified in NVs of HMG diseases**. B) The amino acids in A are shown on sequence alignments of the human SOX members. C) Proposed docking between the conserved amino acids on Sry (yellow) and the CTE of AR (red). D) MD simulations of the CTE interacting with DNA and SRY (left) or Sox9 (right) either docked (blue), AR mutated to all alanines (green) or pulled away from the interaction and free in movement (red).

**Figure 5:**
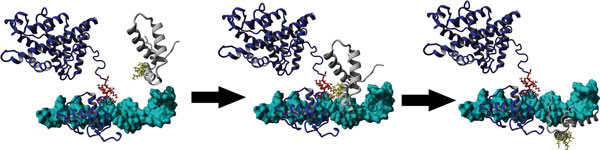
**Recruitment to DNA (cyan) through the interaction of the AR's (blue) C-terminal extension sequence (red) with the identified residues (yellow) of SRY (gray)**.

As an example to illustrate the usefulness of these kinds of studies to SRY protein function, we investigated the possible interactions of SRY with the androgen receptor (AR). For many years we have known that a functional AR is needed for the blood pressure increase due to the spontaneously hypertensive rat (SHR) Y chromosome [21], and we believe this may be through a direct interaction of AR and Sry. The HMG box of Sry [22] or Sox9 [23] is known to directly interact with the AR C-terminal extension (CTE). Examining 75 mammalian Sry sequences we identified amino acids that do not appear to contribute to either DNA interaction or proper folding of the HMG box and yet are highly conserved (Figure 4A yellow). These amino acids were highly conserved across the human Sox members (Figure 4B) and indicate a functional importance that is not related to folding or DNA binding. When docking the CTE sequence to these amino acids, a stable interaction with SRY and Sox9 can be predicted (Figure 4C-D). The binding energy of this is higher than other docking experiments and can possibly take on the fold orientation as shown with modeling approached. Sox9 has high conservation of these amino acids when looking at multiple sequence alignments. The methionine conserved in SRY contributed to the mutated CTE stability (docked As in green of Figure 4D) by enhancing hydrophobic packing missing from Sox9. Interactions between HMGB1/2 and steroid receptors are known to increase recruitment of the steroid receptors to DNA and are dependent on the CTE [24,25]. We propose that this interaction between the CTE of AR and Sry facilitates recruitment to DNA as shown in Figure 5.

## Conclusions

Molecular dynamic simulations support functional conservation for DNA binding and structure of the 8 HMG conserved NVs. Most NVs identified were conserved in the Sox subfamily of HMG proteins. Of these amino acids conserved, amino acids 4 and 29 were identified to have contacts with base pairs of the minor groove contributing to DNA specificity. Several NV amino acids, such as 16 and 45, were not as highly conserved in HMG proteins and likely contribute to individual member specificity. Some Sox conserved amino acids that do not appear to contribute to proper packing or DNA interaction were identified as a potential docking site for interacting with AR. The use of sequences, structures, natural variants in disease phenotypes and molecular dynamics simulations of protein-DNA interaction offers new insights at understanding the HMG domain at an amino acid level. This approach serves as a hypothesis generator for molecular mutagenesis, and protein-protein/protein-DNA interactions.

## Competing interests

The authors declare that they have no competing interests.

## List of abbreviations

NV: natural variant; NVs: natural variants; MD: molecular dynamics; HMG: high mobility group; AR: androgen receptor; PDB: Protein data bank; ps: picosecond; RMSD: root mean square deviation; CTE: C-terminal extension.

## Authors' contributions

JWP performed all alignments, MD simulations, presented data, and compiled the manuscript. TL advised on the biochemistry, structural modeling and structure alignments. ZHD aided in MD simulation setup. AM advised on HMG basics and Sry biology. All authors contributed extensive revisions to both the presentation at the conference and the manuscript; approving both.

## Supplementary Material

Additional file 1 Pages 1-2: Accession codes for all protein sequences used. Page 3: Table 1 Natural Variants in HMG box proteins. Page 4-5: Figure S1 Sequence alignments from 38 Mammalian Sry sequences, Page 6-7: Figure S2 Sequence alignments from multiple species of Sox9. Page 8: Figure S3 Autodock experiment showing the favored confirmation of the AR CTE with the conserved amino acids of Sry. Page 9: Figure S4 Mustang alignment of the CTE confirmation in docking experiments shown on a model of the AR done with I-TASSER. Page 10-12: Additional references in Table 1. Click here for file
