# Supplementary material for: Amino acid function and docking site prediction through combining disease variants, structure alignments, sequence alignments, and molecular dynamics: a study of the HMG domain
Source: BMC Bioinformatics. 2012 Mar 13;13(Suppl 2):S3. doi: 10.1186/1471-2105-13-S2-S3 (PMC3402923; doi:10.1186/1471-2105-13-S2-S3)
Supplement: Additional file 1 — Pages 1-2: Accession codes for all protein sequences used. Page 3: Table 1 Natural Variants in HMG box proteins. Page 4-5: Figure S1 Sequence alignments from 38 Mammalian Sry sequences, Page 6-7: Figure S2 Sequence alignments from multiple species of Sox9. Page 8: Figure S3 Autodock experiment showing the favored confirmation of the AR CTE with the conserved amino acids of Sry. Page 9: Figure S4 Mustang alignment of the CTE confirmation in docking experiments shown on a model of the AR done with I-TASSER. Page 10-12: Additional references in Table 1. [file 1471-2105-13-S2-S3-S1.pdf]

Accession codes:

Swiss-Prot HMG proteins: Q8WY36, Q96RK0, O60381, Q9NP66, Q9P0W2, Q3SYE8, P0C6E5, P09429, P26583, O15347, Q8WW32, Q9UGV6, Q12766, Q9UGU5, Q9UJU2, O96028, Q86U86, P54277, Q969G3, P23497, Q08945, P21675, P36402, Q9HCS4, Q9NQB0, Q00059, Q96NM4, O15405, O94842, O94900, P17480, P0CB47, P0CB48, and O75717;

Swiss-Prot Human Sox proteins: Q05066, P41225, O00570, P48431, Q06945, P35711, P35712, Q9BT81, P57073, P48436, P56693, P35716, O15370, Q9UN79, 4759162, O60248, Q9H6I2, P35713, Q9Y651, 30581117;

Swiss-Prot Mammalian Sry proteins: Q863D9, Q6T723, Q6T724, Q6TC44, Q863C2, Q864R2, Q864R1, Q864R0, Q864Q9, Q8SPQ2, Q27949, Q7JGF9, Q7JGF7, Q8SPQ1, Q9XS37, Q03255, Q69FB1, P51501, Q6TC43, Q9XT60, Q03256, Q863C0, Q9BH08, Q6TC37, Q864P9, Q864Q3, Q863B7, Q6TC27, Q6TC30, Q864Q7, Q6TC46, Q6TC50, Q864P4, P48046, Q864P7, P36390, P36389, Q05066, Q6TC40, Q28447, Q864Q5, Q864P8, Q6TC28, Q9BG90, Q864Q8, Q864Q4, Q6TC39, Q6TC38, Q6TC41, Q67EX7, Q67EX6, Q05738, Q62565, Q62563, Q864Q1, Q864P3, Q864P5, Q6TC36, Q28778, Q28798, Q864P6, Q6TC32, Q6TC31, Q864Q2, Q6TC33, Q864Q6, P36393, Q28783, Q6TC34, P36394, Q03257, P36395, Q864Q0, P36396, Q6TC45;

Swiss-Prot Sox8 multispecies proteins (invertebrates and vertebrates): P57073, Q04886, P57074, Q6VVD7, Q6IZ48, Q52W08, Q5BM60, E6ZGK0, Q38JF0, Q38PQ8, E2BS23, E2B1P3, D3ZR96, E2B1P2, E2R0K1, C3VV12;

Swiss-Prot Sox9 multispecies proteins (invertebrates and vertebrates): P48436, Q6F2E7, Q04887, B7ZR65, Q7YRJ7, P61753, Q9BG89, Q9BG91, P61754, P48434, O18896, Q8JIP0, Q9DE28, Q91WY3, Q0PV97, Q9YGP7, B0G0U8, Q0GA74, D0PQH0, D0PQH1, A8VM83, Q9DE39, A8VM79, Q9IB79, Q5EPK7,

Q08G17, C7B5N9, C7E9Q3, Q8UWL6, C6KJ53, D0PQG9, A8WDX1, C0HAA6, D9DB12, B4XTT2, Q9MYY4, Q9BG92, O73842, Q2I8Y1, Q6B7J1, Q3ZN09, B2WSC3, Q1HQD4, B5ATG2, E2G4I5, A8TT17, B0LT39, B0LT40, B0LT41, Q7T1L4, A9YYK0, A4GNL7, Q6QQQ0, Q2NM26, Q2NM27, B0M178, D1LHP1, A0A8J7, D0VEC6, C0LEW2, D0VXZ7;

Swiss-Prot Sox10 multispecies proteins (invertebrates and vertebrates): P56693, Q04888, Q8AXX8, O55170, Q9W757, A5A763, A4IIJ8, Q90XD1, Q08G18, B7PYW8, Q1I0U0, D1LHP2, Q06FD9;

Swiss-Prot HMGB1 multispecies proteins (invertebrates and vertebrates): P09429, P63158, P63159, P10103, Q7SZ42, Q6YKA4, Q08IE6, B0CM99, A9RA84, P12682, B1MTB0, P36194, Q4R844]

PDB HMG proteins: 2e6o, 2lef, 1gt0, 1o4x, 1hrz, 1j46, 2gzk, 2yul, 3f27, 1i11, 1wz6, 2crj, 1cg7, 1j5n, 1j3d, 1e7j, 1wxl

PDB Sox-specific proteins: 1j46, 1gto, 104x, 2gzk (Sry HMG domain only), 2yul, 3f27, 1i11

Amino Acid	Normal	NV	Protein	Disease	Reference	Classification of Conservation	HMG box	%	Sox proteins	%	Sry proteins	%
1	R	G	Sry	45,X/46,X psu dic (Y)	[26]	Polar Basic	-	**-**	20	**100**	-	**-**
2	V	L	Sry	GDXY	[27]	Hydrophobic	57	**82.609**	20	**100**	75	**100**
2	V	A	Sry	GDXY	[28]							
4	R	G	Sry	GDXY	[29]	Polar Basic	52	**75.362**	20	**100**	75	**100**
4	G	R	HMGB1	GCCL	[20]	Glycine	6	**8.6957**				
5	P	L	Sox-9	CMD1	[30]	Proline	55	**79.71**	20	**100**	75	**100**
6	M	I	Sry	GDXY	[27]	Hydrophobic	64	**92.754**	20	**100**	75	**100**
6	M	R	Sry	GDXY	[31]							
9	F	V	Sry	GDXY	[31]	Hydrophobic / Aromatic	65	**94.203**	20	**100**	75	**100**
9	F	L	Sox-9	CMD1	[32]							
9	F	S	Sox-9	CMD1	[33]							
10	I	T	Sry	GDXY	[34]	Hydrophobic	58	**84.058**	20	**100**	75	**100**
12	W	R	Sox-18	HLTS	[35]	Hydrophobic / Aromatic	68	**98.551**	20	**100**	75	**100**
16	A	V	Sox-9	CMD1	[32] and [19]	Alanine	3	**4.3478**	3	**15**	0	**0**
18	R	S	Sry	GDXY, SR	[36]	Polar Basic	22	**31.884**	19	**95**	75	**100**
20	M	T	Sry	GDXY	[29]	Hydrophobic	58	**84.058**	20	**100**	75	**100**
21	A	P	Sox-18	HLTS	[35]	Hydrophobic	40	**57.971**	20	**100**	75	**100**
29	N	Y	Sry	GDXY, SR	[37]	N or Q	44	**63.768**	20	**100**	75	**100**
31	E	K	Sry	GDXY	[38]	Polar Acidic	55	**79.71**	18	**90**	75	**100**
32	I	M	Sry	GDXY, TH	[39]	Hydrophobic (no M)	63	**91.304**	20	**100**	75	**100**
33	S	G	Sry	GDXY, SR	[40]	S or T	44	**63.768**	20	**100**	75	**100**
33	S	T	Sox-10	YDBHS	[41]							
34	K	M	Sry	SR	[42]	Polar Basic	56	**81.159**	19	**95**	75	**100**
36	L	W	Sry	TS	[Swiss-Prot: D0VTX0]	Hydrophobic	57	**82.609**	20	**100**	75	**100**
37	G	E	Sry	GDXY	[43]	Glycine	41	**59.42**	20	**100**	75	**100**
37	G	R	Sry	GDXY	[44] and [38]							
40	W	R	Sox-9	CMD1	[30]	Hydrophobic / Aromatic	68	**98.551**	20	**100**	75	**100**
43	L	H	Sry	GDXY	[45]	Hydrophobic	64	**92.754**	20	**100**	75	**100**
45	A	E	HMGB1	GCCL	[20]	Hydrophobic	14	**20.29**	3	**15**	1	**1.3333**
48	K	I	Sry	GDXY	[39]	Polar Basic (with also Glutamine)	69	**100**	20	**100**	75	**100**
49	R	P	Sox-9	CMD1	[30]	Polar Basic (with also Glutamine)	37	**53.623**	16	**80**	22	**29.333**
50	P	R	Sry	GDXY	[46]	Proline	26	**37.681**	20	**100**	75	**100**
51	F	S	Sry	GDXY	[47]	Hydrophobic / Aromatic	66	**95.652**	20	**100**	75	**100**
51	F	L	Sox-9	CMD1	[48]							
55	A	T	Sry	GDXY	[49]	Hydrophobic	59	**85.507**	17	**85**	75	**100**
55	A	T	Sox-9	CMD1	[48]							
60	A	P	Sry	SR	[42]	Hydrophobic	28	**40.58**	16	**80**	70	**93.333**
62	H	Y	Sox-9	CMD1	[19]	Polar Basic	25	**36.232**	20	**100**	75	**100**
67	P	L	Sry	GDXY, SR	[40]	Proline	27	**39.13**	20	**100**	74	**98.667**
67	P	R	Sox-9	CMD1	[30]							
69	Y	C	Sry	GDXY, SR	[50]	Hydrophobic / Aromatic	57	**82.609**	20	**100**	74	**98.667**
69	Y	F	Sry	GDXY	[51]							
69	Y	I	Sry	SR	[Swiss-Prot: D0VTX2]							
73	P	R	Sry	GDXY, SR	[52]	Proline	-		20	**100**	-	**-**
75	R	W	Sry	GDXY	[29]	Polar Basic	-		16	**80**	-	**-**
Total							**69**		**20**		**75**	

Table 1 Natural Variants (NVs) in HMG box proteins. Diseases: 45,X/46,X psu dic (Y)= mosaic sex karyotype, GDXY=gonadal dysgenesis (XY Female), GCCL=gastric-carcinoma cell line, CMD1=campomelic dysplasia, HLTS=hypotrichosis-lymphedema-telangiectasia, SR=sex reversal, TH= true hermaphroditism, YDBHS= Yemenite deaf-blind hypopigmentation syndrome, and TS= Turner syndrome. Classification of Conservation: Polar Basic=K,R,H; Hydrophobic=V,L,I,F,W,M,P; Hydrophobic/Aromatic=F,W,Y,H; Polar Acidic=D,E. Percent conservation is based on the number to the left divided by the total at the bottom.

Figure S1

```
Homo_sapiens_A7WPU8_                GENSKGNVQDRVKRPMNAFIVWSRDQRRKMALENPRMRNSEISKQLGYQW 98
Gorilla_gorilla_784985|emb|CAA      GENSKGSVQDRVKRPMNAFIVWSRDQRRKMALENPRMRNSEISKQLGYQW 98
Pan_troglodytes_Q28798_             GENSKDSVQDRVKRPMNAFFVWSRDQRRKMALENPRMRNSEISKQLGYQW 98
Macaca_fascicularis_11344669|g      GENSKGSVQDKVKRPMNAFIVWSRDQRRKMALENPKMRNSEISKQLGYQW 98
Macaca_mulatta_11344685|gb|AAG      GENSKGSVQDKVKRPMNAFIVWSRDQRRKMALENPKMRNSEISKQLGYQW 98
Callithrix_aurita_20563186|gb|      GENSKGSVQNRVKRPMNAFIVWSRDQRRKMAVENPQMRNSEISKRLGYQW 98
Callithrix_jacchus_20563160|gb      GENSKGSVQNRVKRPMNAFIVWSRDQRRKMAVENPQMRNSEISKRLGYQW 98
Callimico_goeldii_20563168|gb|      GENSKGSVQNRVKRPMNAFIVWSRDQRRKMAVENPQMRNSEISKRLGYQW 98
Saguinus_midas_midas_20563184|      RENSKGSVQNRVKRPMNAFIVWSRDQRRKMAVENPQMRNSEISKRLGYQW 98
Leontopithecus_rosalia_2056314      GENSKGSVQNRVKRPMNAFIVWSRDQRRKMAVENPQMRNSEISKRLGYQW 98
Cebus_xanthosternos_20563182|g      GENSKGNVQNRVKRPMNAFIVWSRDQRRKMAVENPQMRNSEISKRLGYQW 98
Cebus_olivaceus_20563180|gb|AA      GENSKGNVQNRVKRPMNAFIVWSRDQRRKMAVENPQMRNSEISKRLGYQW 98
Aotus_infulatus_20563154|gb|AA      GENSKDSVQNRVKRPMNAFIVWSRDQRRKMAVENPQMRNSEISKRLGYQW 98
Aotus_azarai_20563152|gb|AAM27      GENSKDSVQNRVKRPMNAFIVWSRDQRRKMAVENPQMRNSEISKRLGYQW 98
Bos_taurus_B6S2A2_                  GEHVRESSQDHVKRPMNAFIVWSRERRRKVALENPKMKNSDISKQLGYEW 92
Bos_grunniens_210063367|gb|ACJ      GEHVRESSQDHVKRPMNAFIVWSRERRRKVALENPKMKNSDISKQLGYEW 92
Bubalus_bubalis_71082650|gb|AA      GENVRESSQDHIKRPMNAFILWSREGRRKLALENPKMKNSEISKQLGYEW 92
Hydropotes_inermis_118490115|g      GEIVRESGQDHVKRPMNAFLVWSRERRRKVALENPKMQNSEISKQLGYEW 91
Capreolus_pygargus_tianschanic      GENVRESGQDRVKRPMNAFIVWSRERRRKVALENPNMQNSEISKQLGYEW 91
Cervus_elaphus_canadensis_1184      GENVRESGQDHVKRPMNAFIVWSRERRRKVALENPEMQNSEISKQLGYEW 92
Balaenoptera_acutorostrata_612      GGNGRESGQDRVKRPMNAFIVWSRDQRRKVALENPQMQNSEISKRLGYDW 92
Balaenoptera_bonaerensis_Q864R      GGNGRESGQDRVKRPMNAFIVWSRDQRRKVALENPQMQNSEISKRLGYDW 92
Physeter_catodon_Q864Q6_            GGNGRESGQDRVKRPMNAFIVWSRDQRRKVALENPQMQNSEISKRLGYDW 92
Sus_scrofa_P36393_                  RGSGRESGQDRVKRPMNAFIVWSRDQRRKVALENPQMQNSEISKWLGCKW 10
Panthera_leo_77176743|gb|ABA64      RGNGRDRGQDRVKRPMNAFMVWSRDQRRKVALENPQTQNSEISKQLGYQW 92
Panthera_tigris_77176735|gb|AB      RGSGRDRGQDRVKRPMNAFMVWSRDQRRKVALENPQTQNSEISKQLGYQW 92
Acinonyx_jubatus_77176759|gb|A      GGKGRDRGQDRVKRPMNAFMVWSRDQRRKVALENPQTQNSEISKQLGYQW 92
Zalophus_californianus_3804910      RGNGRDSGQNRVRRPMNAFMVWSRDQRRKVALENPQMQNSEISKQLGYQW 92
Eumetopias_jubatus_38049105|gb      RGNGRDSGQNRVRRPMNAFMVWSRDQRRKVALENPQMQNSEISKQLGYQW 92
Ursus_arctos_61252072|sp|P3639      EGNSRDSGQNRVRRPMNAFMLWSRDQRRKVALENPQMQNSEISKQLGYQW 92
Ursus_maritimus_160960124|emb|      EGNSRDSGQNRVRRPMNAFMLWSRDQRRKVALENPQMQNSEISKQLGYQW 92
Globicephala_macrorhynchus_150      GGNCRESGQDRVKRPMNAFIVWSRDQRRKVALENPQMQNSEISKRLGYDW 92
Tursiops_truncatus_Q864Q0_          GGNCRESGQDRVKRPMNAFIVWSRDQRRKVALENPQMQNSEISKRLGYDW 92
Delphinapterus_leucas_Q864Q3_       GGNCRESGQDRVKRPMNAFIVWSRDQRRKVALENPQMQNSEISKRLGYDW 92
Phocoenoides_phocoena_Q864Q2_       GGNCRESGQDRVKRPMNAFIVWSRDQRRKVALENPQMQNSEISKRLGYDW 92
Equus_caballus_P36389_              RGNGRENGQDRVKRPMNAFMVWSRDHRRKVALENPQLQNSEISKRLGCQW 92
Mus_musculus_Q05738_                -------MEGHVKRPMNAFMVWSRGERHKLAQQNPSMQNTEISKQLGCRW 43
Rattus_norvegicus_Sry1              -------MEGHVKRPMNAFMVWSRGERRKLAQQNPSMQNSEISKHLGYQW 43
                                            :.:::******::***  ::*:* :**  :*::*** **  *
```

Figure S1 Sequence alignments of 38 of the Mammalian Sry sequences showing conservation of the natural variant amino acids (Purple, red, and cyan) and the identified amino acids that may be interacting with androgen receptor (yellow).

```
Homo_sapiens_A7WPU8_                      KMLTEAEKWPFFQEAQKLQAMHREKYPNYKYRPRRKAKMLPKNCSLLPAD
Gorilla_gorilla_784985|emb|CAA            KMLTEAEKWPFFQEAQKLQAMHREKYPNYKYRPRRKAKMLPKNCSLLPAD
Pan_troglodytes_Q28798_                   KMLTEAEKWPFFQEAQKLQAMHREKYPNYKYRPRRKANMLPKNCSLLPAD
Macaca_fascicularis_11344669|g            KMLTEADKWPFFQEAQKLQAMHREKYPNYKYRPRRKAKMLQNSCSLLPAD
Macaca_mulatta_11344685|gb|AAG            KMLTEADKWPFFQEAQKLQAMHREKYPNYKYRPRRKAKMLQNSCSLLPAD
Callithrix_aurita_20563186|gb|            KLLTEAEKWPFFQEAQKLQAMHREKYPNYKYRPRRKANMLQNNDSLLTAD
Callithrix_jacchus_20563160|gb            KLLTEAEKWPFFQEAQKLQAMHREKYPNYKYRPRRKANMLQNNDSLLTAD
Callimico_goeldii_20563168|gb|            KLLTEAEKWPFFQEAQKLQAMHREKYPNYKYRPRRKANMLQNNDSLLTAN
Saguinus_midas_midas_20563184|            KLLTEAEKWPFFQEAQKLQAMHREKYPNYKYRPRRKANMLQNN-SLLTAD
Leontopithecus_rosalia_2056314            KLLTEAEKWPFFQEAQKLQAMHREKYPNYKYRPRRKANMLQNNDSLLNAD
Cebus_xanthosternos_20563182|g            KLLSEAEKWPFFQEAQKLQAMHREKYPNYKYRPRRKANLLQNDDSLRPAD
Cebus_olivaceus_20563180|gb|AA            KLLTEAEKWPFFQEAQKLQAMHREKYPNYKYRPRRKANLLQNNDSLRPAD
Aotus_infulatus_20563154|gb|AA            KLLTEAEKWPFFQEAQKLQAMHREKYPNYKYRPRRKANVLQNNESFLPAD
Aotus_azarai_20563152|gb|AAM27            KLLTEAEKWPFFQEAQKLQAMHREKYPNYKYRPRRKANVLQNNESFLPAD
Bos_taurus_B6S2A2_                        KRLTDAEKRPFFEEAQRLLAIHRDKYPGYKYRPRRRAK---RPQKSLPAD
Bos_grunniens_210063367|gb|ACJ            KRLTDAEKRPFFEEAQRLLAIHRDKYPGYKYRPRRRAK---RPQKSLPAD
Bubalus_bubalis_71082650|gb|AA            KRLTDAEKRPFFEEAQRLLSIHRDKYPGYKYRPRRKAK---RLQKSLPAD
Hydropotes_inermis_118490115|g            KRLTAAEKRPFFEEAQRLLAIHRDKYPGYKYRPRRKTK---RQQKLLPAD
Capreolus_pygargus_tianschanic            KRLTDAEKRPFFEEAQRLLAIHRDKYPGYKYRPRRKTK---RQQKLLPAD
Cervus_elaphus_canadensis_1184            KRLTDAEKRPFFEEAQRLLAVHRDKYPGYKYRPRRKTK---RQQKLLPAD
Balaenoptera_acutorostrata_612            KMLTEAEKQPFFEEAQRLRAMHRDKYPGYKYRPRRKAK---RPQKLLPAD
Balaenoptera_bonaerensis_Q864R            KMLTEAEKQPFFEEAQRLRAMHRDKYPGYKYRPRRKAK---RPQKLLPAD
Physeter_catodon_Q864Q6_                  KMLTEAEKQPFFEEAQRLRAMHRDKYPGYKYHPRRKPK---RPQESLPAD
Sus_scrofa_P36393_                        KMLTEAEKRPFFEEAQRLQAVHRDKYPGYKYRPRRKGE---RAQNLLPAE
Panthera_leo_77176743|gb|ABA64            KMLTEAEKWPFFEEAQRLQALHREKYPGYKYRPRRKATPEKSDKLLPAE
Panthera_tigris_77176735|gb|AB            KMLTEAEKWPFFEEAQRLQALHREKYPGYKYRPRRKATPEKSDKLLPAE
Acinonyx_jubatus_77176759|gb|A            KMLTEAEKWPFFEEAQRLQALHREKYPGYKYRPRRKATPEKSDKLLPAD
Zalophus_californianus_3804910            KMLTEAEKWPFFEEAQRLQAVHREKYPDYKYRPRRKALPQKSDKLLPAA
Eumetopias_jubatus_38049105|gb            KMLTEAEKWPFFEEAQRLQAVHREKYPDYKYRPRRKALPQKSDKLLPAA
Ursus_arctos_61252072|sp|P3639            EMLTEAEKWPFFEEAQRLQAMHRQKYPDYKYRPRRKATPQKDDKLLPSA
Ursus_maritimus_160960124|emb|            KMLTEAEKWPFFEEAQRLQAMHRQKYPDYKYRPRRKATPQKDDKLLPSA
Globicephala_macrorhynchus_150            KMLTEAEKQPFFEEAQRLRAMHRDKYPGYKYRPRRKAKEATEIASRRLFS
Tursiops_truncatus_Q864Q0_                KMLTEAEKQPFFEEAQRLRAMHRDKYPGYKYRPRRKAKEATEIASRRLFS
Delphinapterus_leucas_Q864Q3_             KMLTEAEKQPFFEEAQRLRAMHRDKYPGYKYRPRRKAKEATEIASRRLFS
Phocoenoides_phocoena_Q864Q2_             KMLTEAEKQPFFEEAQRLRAMHRDKYPGYKYRPRRKAKEATEIASRRLFS
Equus_caballus_P36389_                    KMLTEAEKLPFFEEAQRLRAMHQEKYPDYKYRPRRKAKMPQKSDKPLPQT
Mus_musculus_Q05738_                      KSLTEAEKRPFFQEAQRLKILHREKYPNYKYQPHRRAKVSQRSGILQPAV
Rattus_norvegicus_Sry1                    KSLTEAEKRPFFQEAQRLKTLHREKYPNYKYQPHRRVKVPQRSYTLQREV
                                          : *: *:* ***:***:*  :*::***.***:*::       .
```

Figure S1 (Cont.) Sequence alignments of 38 of the Mammalian Sry sequences showing conservation of the natural variant amino acids (Purple, red, and cyan) and the identified amino acids that may be interacting with androgen receptor (yellow).

Figure S2

```
sp|Q6F2E7|105-173_Xenopus_trop      VKRPMNAFMVWAQAARRKLADQYPHLHNAELSKTLGKLWRLLNEGEKRPF 50
Q9IB79_Glandirana_rugosa            VKRPMNAFMVWAQAARRKLADQYPHLHNAELSKTLGKLWRLLNETEKRPF 50
Q0GA74_Bufo_bufo                    VKRPMNAFMVWAQAARRKLADQYPHLHNAELSKTLGKLWRLLNESEKRPF 50
C0LEW2_Bufo_marinus                 VKRPMNAFMVWAQAARRKLADQYPHLHNAELSKTLGKLWRLLNESEKRPF 50
sp|P48436|105-173_Homo_sapiens      VKRPMNAFMVWAQAARRKLADQYPHLHNAELSKTLGKLWRLLNESEKRPF 50
A8WDX1_Trachemys_scripta_elega      -KRPMNAFMVWAQAARRKLADQYPHLHNAELSKTLGKLWRLLNESEKRPF 49
sp|Q7YRJ7|105-173_Canis_famili      VKRPMNAFMVWAQAARRKLADQYPHLHNAELSKTLGKLWRLLNESEKRPF 50
sp|Q9BG91|105-173_Callithrix_j      VKRPMNAFMVWAQAARRKLADQYPHLHNAELSKTLGKLWRLLNESEKRPF 50
sp|O18896|105-173_Sus_scrofa        VKRPMNAFMVWAQAARRKLADQYPHLHNAELSKTLGKLWRLLNESEKRPF 50
C7B5N9_Crocodylus_palustris         VKRPMNAFMVWAQAARRKLADQYPHLHNAELSKTLGKLWRLLNESEKRPF 50
Q9BG92_Hylobates                    VKRPMNAFMVWAQAARRKLADQYPHLHNAELSKTLGKLWRLLNESEKRPF 50
B0LT41_Nyctereutes_procyonoide      VKRPMNAFMVWAQAARRKLADQYPHLHNAELSKTLGKLWRLLNESEKRPF 50
D1LHP1_Monodelphis_domestica        VKRPMNAFMVWAQAARRKLADQYPHLHNAELSKTLGKLWRLLNESEKRPF 50
sp|Q04887|105-173_Mus_musculus      VKRPMNAFMVWAQAARRKLADQYPHLHNAELSKTLGKLWRLLNESEKRPF 50
O73842_Calotes_versicolor           VKRPMNAFMVWAQAARRKLADQYPHLHNAQLSKTLGKLWRLLNESEKRPF 50
sp|P61753|105-173_Macaca_mulat      VKRPMNAFMVWAQAARRKLADQYPHLHNAELSKTLGKLWRLLNESEKRPF 50
sp|P61754|105-173_Pongo_pygmae      VKRPMNAFMVWAQAARRKLADQYPHLHNAELSKTLGKLWRLLNESEKRPF 50
Q9DE28_Eublepharis_macularius       VKRPMNAFMVWAQAARRKLADQYPHLHNAELSKTLGKLWRLLNESEKRPF 50
C7E9Q3_Lepidochelys_olivacea        VKRPMNAFMVWAQAARRKLADQYPHLHNAELSKTLGKLWRLLNESEKRPF 50
B0LT39_Vulpes_vulpes                VKRPMNAFMVWAQAARRKLADQYPHLHNAELSKTLGKLWRLLNESEKRPF 50
Q2NM26_Coturnix_coturnix            VKRPMNAFMVWAQAARRKLADQYPHLHNAELSKTLGKLWRLLNESEKRPF 50
A0A8J7_Trimeresurus_flavovirid      VKRPMNAFMVWAQAARRKLADQYPHLHNAELSKTLGKLWRLLNESEKRPF 50
D0VXZ7_Leiolepis_reevesii           VKRPMNAFMVWAQAARRKLADQYPHLHNAELSKTLGKLWRLLNESEKRPF 50
Q2NM27_Anas_platyrhynchos           VKRPMNAFMVWAQAARRKLADQYPHLHNAELSKTLGKLWRLLNESEKRPF 50
B0LT40_Vulpes_lagopus               VKRPMNAFMVWAQAARRKLADQYPHLHNAELSKTLGKLWRLLNESEKRPF 50
Q9MYY4_Bos_taurus                   VKRPMNAFMVWAQAARRKLADQYPHLHNAELSKTLGKLWRLLNESEKRPF 50
Q9YGP7_Alligator_mississippien      VKRPMNAFMVWAQAARRKLADQYPHLHNAELSKTLGKLWRLLNESEKRPF 50
sp|P48434|106-174_Gallus_gallu      VKRPMNAFMVWAQAARRKLADQYPHLHNAELSKTLGKLWRLLNESEKRPF 50
Q91WY3_Rattus_norvegicus            VKRPMNAFMVWAQAARRKLADQYPHLHNAELSKTLGKLWRLLNESEKRPF 50
sp|Q9BG89|105-173_Pan_troglody      VKRPMNAFMVWAQAARRKLGDQYPHLHNAELSKTLGKLWRLLNESEKRPF 50
A8VM83_Ctenopharyngodon_idella      -KRPMNAFMVWAQAARRKLADQYPHLHNAELSKTLGKLWRLLNEGEKRPF 49
A8VM79_Mylopharyngodon_piceus       -KRPMNAFMVWAQAARRKLADQYPHLHNAELSKTLGKLWRLLNEGEKRPF 49
D9DB12_Xenocypris_davidi            -KRPMNAFMVWAQAARRKLADQYPHLHNAELSKTLGKLWRLLNEGEKRPF 49
B4XTT2_Carassius_carassius          -KRPMNAFMVWAQAARRKLADQYPHLHNAELSKTLGKLWRLLNEGEKRPF 49
B2WSC3_Megalobrama_amblycephal      -KRPMNAFMVWAQAARRKLADQYPHLHNAELSKTLGKLWRLLNEGEKRPF 49
A9YYK0_Carassius_carassius          -KRPMNAFMVWAQAARRKLADQYPHLHNAELSKTLGKLWRLLNEGEKRPF 49
A4GNL7_Megalobrama_amblycephal      -KRPMNAFMVWAQAARRKLADQYPHLHNAELSKTLGKLWRLLNEGEKRPF 49
Q6QQQ0_Carassius_auratus            -KRPMNAFMVWAQAARRKLADQYPHLHNAELSKTLGKLWRLLNEGEKRPF 49
sp|B7ZR65|105-173_Xenopus_laev      VKRPMNAFMVWAQAARRKLADQYPHLHNAELSKTLGKLWRLLNEGEKRPF 50
Q8JIP0_Oryzias_latipes              VKRPMNAFMVWAQAARRKLADQYPHLHNAELSKTLGKLWRLLNEGEKRPF 50
D0PQH0_Leucoraja_erinacea           VKRPMNAFMVWAQAARRKLADQYPHLHNAELSKTLGKLWRLLNEGEKRPF 50
Q9DE39_Oncorhynchus_mykiss          VKRPMNAFMVWAQAARRKLADQYPHLHNAELSKTLGKLWRLLNEGEKRPF 50
B0G0U8_Oreochromis_aureus           VKRPMNAFMVWAQAARRKLADQYPHLHNAELSKTLGKLWRLLNEGEKRPF 50
D0PQG9_Scyliorhinus_canicula        VKRPMNAFMVWAQAARRKLADQYPHLHNAELSKTLGKLWRLLNEGEKRPF 50
D0PQH1_Acipenser_baerii             VKRPMNAFMVWAQAARRKLADQYPHLHNAELSKTLGKLWRLLNEGEKRPF 50
Q5EPK7_Acipenser_sturio             VKRPMNAFMVWAQAARRKLADQYPHLHNAELSKTLGKLWRLLNEGEKRPF 50
Q08G17_Acipenser_schrenckii         VKRPMNAFMVWAQAARRKLADQYPHLHNAELSKTLGKLWRLLNEGEKRPF 50
C0HAA6_Salmo_salar                  VKRPMNAFMVWAQAARRKLADQYPHLHNAELSKTLGKLWRLLNEGEKRPF 50
B5ATG2_Pleurodeles_waltl            VKRPMNAFMVWAQAARRKLADQYPHLHNAELSKTLGKLWRLLNEGEKRPF 50
E2G4I5_Andrias_davidianus           VKRPMNAFMVWAQAARRKLADQYPHLHNAELSKTLGKLWRLLNEGEKRPF 50
D0VEC6_Sebastiscus_marmoratus       VKRPMNAFMVWAQAARRKLADQYPHLHNAELSKALGKLWRLLNEGEKRPF 50
Q0PV97_Poecilia_reticulata          VKRPMNAFMVWAQAARRKLADQYPHLHNAELSKTLGKLWRLLNEVEKRPF 50
Q8UWL6_Takifugu_rubripes            VKRPMNAFMVWAQAARRKLADQYPHLHNAELSKTLGKLWRLLNEVEKRPF 50
C6KJ53_Epinephelus_coioides         VKRPMNAFMVWAQAARRKLADQYPHLHNAELSKTLGKLWRLLNEVEKRPF 50
Q6B7J1_Epinephelus_akaara           VKRPMNAFMVWAQAARRKLADQYPHLHNAELSKTLGKLWRLLNEVEKRPF 50
Q1HQD4_Odontesthes_hatcheri         VKRPMNAFMVWAQAARRKLADQYPHLHNAELSKTLGKLWRLLNEVEKRPF 50
A8TT17_Cynoglossus_semilaevis       VKRPMNAFMVWAQAARRKLADQYPHLHNAELSKTLGKLWRLLNEVEKRPF 50
Q7T1L4_Odontesthes_bonariensis      VKRPMNAFMVWAQAARRKLADQYPHLHNAELSKTLGKLWRLLNEVEKRPF 50
Q2I8Y1_Petromyzon_marinus           VKRPMNAFMVWAQATRRKLADQYPHLHNAELSKTLGKLWRLLSENEKRPF 50
                                             **::. *:*:..: *::**:::** **  *:**.: **:**
```

Figure S2 (Cont.)

```
sp|Q6F2E7|105-173_Xenopus_trop       VEEAERLRIQHKKDHPDYK 69
Q9IB79_Glandirana_rugosa             VEEAERLRIQHKKDHPDYK 69
Q0GA74_Bufo_bufo                     VEEAERLRIQHKKDHPDYK 69
C0LEW2_Bufo_marinus                  VEEAERLRIQHKKDHPDYK 69
sp|P48436|105-173_Homo_sapiens       VEEAERLRVQHKKDHPDYK 69
A8WDX1_Trachemys_scripta_elega       VEEAERLRVQHKKDHPDYK 68
sp|Q7YRJ7|105-173_Canis_famili       VEEAERLRVQHKKDHPDYK 69
sp|Q9BG91|105-173_Callithrix_j       VEEAERLRVQHKKDHPDYK 69
sp|O18896|105-173_Sus_scrofa         VEEAERLRVQHKKDHPDYK 69
C7B5N9_Crocodylus_palustris          VEEAERLRVQHKKDHPDYK 69
Q9BG92_Hylobates                     VEEAERLRVQHKKDHPDYK 69
B0LT41_Nyctereutes_procyonoide       VEEAERLRVQHKKDHPDYK 69
D1LHP1_Monodelphis_domestica         VEEAERLRVQHKKDHPDYK 69
sp|Q04887|105-173_Mus_musculus       VEEAERLRVQHKKDHPDYK 69
O73842_Calotes_versicolor            VEEAERLRVQHKKDHPDYK 69
sp|P61753|105-173_Macaca_mulat       VEEAERLRVQHKKDHPDYK 69
sp|P61754|105-173_Pongo_pygmae       VEEAERLRVQHKKDHPDYK 69
Q9DE28_Eublepharis_macularius        VEEAERLRVQHKKDHPDYK 69
C7E9Q3_Lepidochelys_olivacea         VEEAERLRVQHKKDHPDYK 69
B0LT39_Vulpes_vulpes                 VEEAERLRVQHKKDHPDYK 69
Q2NM26_Coturnix_coturnix             VEEAERLRVQHKKDHPDYK 69
A0A8J7_Trimeresurus_flavovirid       VEEAERLRVQHKKDHPDYK 69
D0VXZ7_Leiolepis_reevesii            VEEAERLRVQHKKDHPDYK 69
Q2NM27_Anas_platyrhynchos            VEEAERLRVQHKKDHPDYK 69
B0LT40_Vulpes_lagopus                VEEAERLRVQHKKDHPDYK 69
Q9MYY4_Bos_taurus                    VEEAERLRVQHKKDHPDYK 69
Q9YGP7_Alligator_mississippien       VEEAERLRVQHKKDHPDYK 69
sp|P48434|106-174_Gallus_gallu       VEEAERLRVQHKKDHPDYK 69
Q91WY3_Rattus_norvegicus             VEEAERLRVQHRKDHPDYK 69
sp|Q9BG89|105-173_Pan_troglody       VEEAERLRVQHKKDHPDYK 69
A8VM83_Ctenopharyngodon_idella       VEEAERLRVQHKKDHPDYK 68
A8VM79_Mylopharyngodon_piceus        VEEAERLRVQHKKDHPDYK 68
D9DB12_Xenocypris_davidi             VEEAERLRVQHKKDHPDYK 68
B4XTT2_Carassius_carassius           VEEAERLRVQHKKDHPDYK 68
B2WSC3_Megalobrama_amblycephal       VEEAERLRVQHKKDHPDYK 68
A9YYK0_Carassius_carassius           VEEAERLRVQHKKDHPDYK 68
A4GNL7_Megalobrama_amblycephal       VEEAERLRVQHKKDHPDYK 68
Q6QQQ0_Carassius_auratus             VEEAERLRVQHKKDHPDYK 68
sp|B7ZR65|105-173_Xenopus_laev       VEEAERLRVQHKKDHPDYK 69
Q8JIP0_Oryzias_latipes               VEEAERLRVQHKKDHPDYK 69
D0PQH0_Leucoraja_erinacea            VEEAERLRVQHKKDHPDYK 69
Q9DE39_Oncorhynchus_mykiss           VEEAERLRVQHKKDHPDYK 69
B0G0U8_Oreochromis_aureus            VEEAERLRVQHKKDHPDYK 69
D0PQG9_Scyliorhinus_canicula         VEETERLRVQHKEDHPDYK 69
D0PQH1_Acipenser_baerii              VEEAERLRVQHKKDHPDYK 69
Q5EPK7_Acipenser_sturio              VEEAERLRVQHKKDHPDYK 69
Q08G17_Acipenser_schrenckii          VEEAERLRVQHKKDHPDYK 69
C0HAA6_Salmo_salar                   VEEAERLRVQHKKDHPDYK 69
B5ATG2_Pleurodeles_waltl             VEEAERLRVQHKKDHPDYK 69
E2G4I5_Andrias_davidianus            VEEAERLRVQHKKDHPDYK 69
D0VEC6_Sebastiscus_marmoratus        VEEAERLRVQHKKDHPDYK 69
Q0PV97_Poecilia_reticulata           VEEAERLRVQHKKDHPDYK 69
Q8UWL6_Takifugu_rubripes             VEEAERLRVQHKKDHPDYK 69
C6KJ53_Epinephelus_coioides          VEEAERLRVQHKKDHPDYK 69
Q6B7J1_Epinephelus_akaara            VEEAERLRVQHKKDHPDYK 69
Q1HQD4_Odontesthes_hatcheri          VEEAERLRVQHKKDHPDYK 69
A8TT17_Cynoglossus_semilaevis        VEEAERLRVQHKKDHPDYK 69
Q7T1L4_Odontesthes_bonariensis       VEEAERLRVQHKKDHPDYK 69
Q2I8Y1_Petromyzon_marinus            VEEAERLRVQHKKDHPDYK 69
                                     :::::***  * :::***
```

Figure S2 (Pages 6-7) Sequence alignments of Sox9 sequences showing amino acid 16 (green) and the conservation of the amino acids interacting with androgen receptor (yellow).

Figure S3

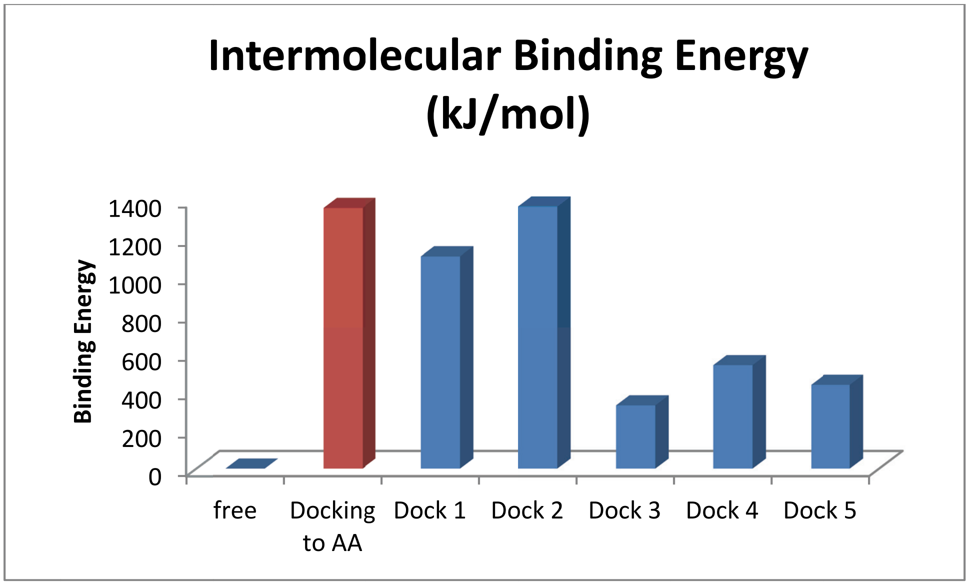

Figure S3 Binding energies of the AR CTE segment either free, in the form proposed to interact with the conserved amino acids of Sry (Docking to AA, red), or five predictions of docking sites using AutoDock. Dock1 and Dock2 produced similar energy as the proposed interaction. Dock 1 was located close to the predicted site of conservation and may serve some role in interaction but with lower energy of formation, while dock2 fell outside the HMG box and thus not relative to known interaction sites (that the interaction occurs in the HMG box).

Figure S4

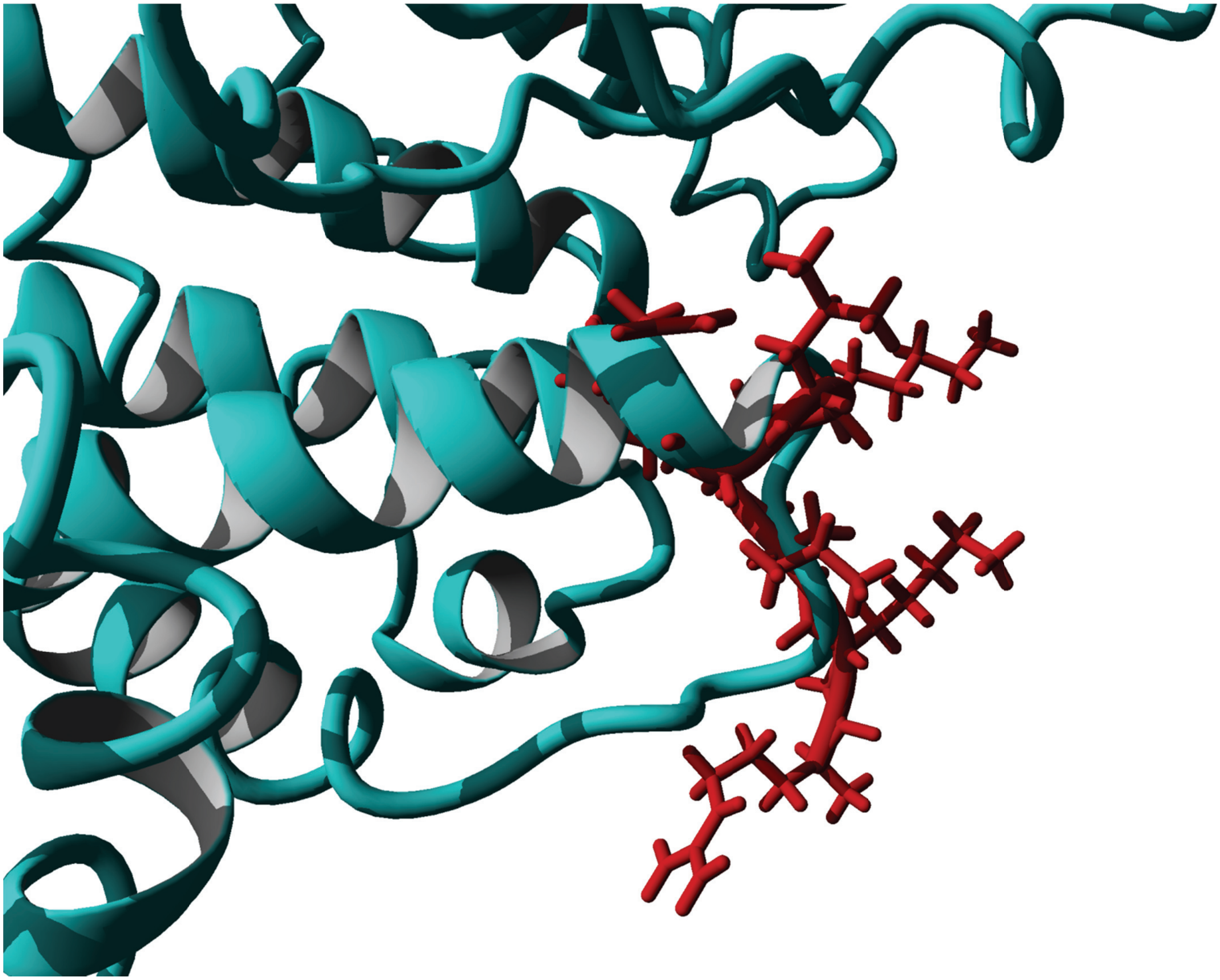

Figure S4 Alignment of the AR CTE segment in the confirmation of its docking to Sry and DNA aligned with a model created of the full AR (RMSD of 1.253Å over 7 amino acids) using I-TASSER. High homology in structure predicts an increase likelihood that the folding seen in docking studies of the CTE may be found in nature that favors the interaction with the Sry site.

Additional References:

26. Fernandez R, Marchal JA, Sanchez A, Pasaro E: **A Point mutation, R59G, within the HMG-SRY box in a female 45,X/46,X, psu dic(Y).** *Hum Genet* 2002, **111**:242-246.

27. Berta P, Hawkins JR, Sinclair AH, Taylor A, Griffiths BL, Goodfellow PN, Fellous M: **Genetic evidence equating SRY and the testis-determining factor**. *Nature* 1990, **348**:448-450.

28. Hiort O, Gramss B, Klauber GT: **True hermaphroditism with 46,XY karyotype and a point mutation in the *SRY* gene**. *J Pediatr* 1995, **126**:1022.

29. Affara NA, Chalmers IJ, Ferguson-Smith MA: **Analysis of the SRY gene in 22 sex-reversed XY females identifies four new point mutations in the conserved DNA binding domain**. *Hum Mol Genet* 1993, **2**:785-789.

30. Meyer J, Südbeck P, Held M, Wagner T, Schmitz ML, Bricarelli FD, Eggermont E, Friedrich U, Haas OA, Kobelt A, Leroy JG, Van Maldergem L, Michel E, Mitulla B, Pfeiffer RA, Schinzel A, Schmidt H, Scherer G: **Mutational analysis of the SOX9 gene in campomelic dysplasia and autosomal sex reversal: lack of genotype/phenotype correlations**. *Hum Mol Genet* 1997, **6**:91-98.

31. Scherer G, Held M, Erdel M, Meschede D, Horst J, Lesniewicz R, Midro AT: **Three novel SRY mutations in XY gonadal dysgenesis and the enigma of XY gonadal dysgenesis cases without SRY mutations**. *Cytogenet Cell Genet* 1998,**80**:188-192.

32. Kwok C, Weller PA, Guioli S, Foster JW, Mansour S, Zuffardi O, Punnett HH, Dominguez-Steglich MA, Brook JD, Young ID, Goodfellow PN, Schafer AJ: **Mutations in SOX9, the gene responsible for Campomelic dysplasia and autosomal sex reversal**. *Am J Hum Genet* 1995, **57**:1028-1036.

33. Goji K, Nishijima E, Tsugawa C, Nishio H, Pokharel RK, Matsuo M: **Novel missense mutation in the HMG box of SOX9 gene in a Japanese XY male resulted in campomelic dysplasia and severe defect in masculinization**. *Hum Mutat* 1998, (Suppl 1):S114-116.

34. Haqq CM, King CY, Ukiyama E, Falsafi S, Haqq TN, Donahoe PK, Weiss MA: **Molecular basis of mammalian sexual determination: activation of Müllerian inhibiting substance gene expression by SRY**. *Science* 1994, **266**:1494-1500.

35. Irrthum A, Devriendt K, Chitayat D, Matthijs G, Glade C, Steijlen PM, Fryns JP, Van Steensel MA, Vikkula M: **Mutations in the transcription factor gene SOX18 underlie recessive and dominant forms of hypotrichosis-lymphedema-telangiectasia.** *Am J Hum Genet* 2003, **72**:1470-1478.

36. Imai A, Takagi A, Tamaya T: **A novel sex-determining region on Y (SRY) missense mutation identified in a 46,XY female and also in the father**. *Endocr J* 1999, **46**:735-739.

37. Okuhara K, Tajima T, Nakae J, Fujieda K: **A novel missense mutation in the HMG box region of the SRY gene in a Japanese patient with an XY sex reversal**. *J Hum Genet*. 2000, **45**:112-114.

38. Cunha JL, Soardi FC, Bernardi RD, Oliveira LE, Benedetti CE, Guerra-Junior G, Maciel-Guerra AT, de Mello MP: **The novel p.E89K mutation in the SRY gene inhibits DNA binding and causes the 46,XY disorder of sex development**. *Braz J Med Biol Res* 2011, **44**:361-365.

39. Hawkins JR, Taylor A, Goodfellow PN, Migeon CJ, Smith KD, Berkovitz GD: **Evidence for increased prevalence of SRY mutations in XY females with complete rather than partial gonadal dysgenesis**. *Am J Hum Genet* 1992, **51**:979-984.

40. Schmitt-Ney M, Thiele H, Kaltwasser P, Bardoni B, Cisternino M, Scherer G: **Two novel SRY missense mutations reducing DNA binding identified in XY females and their mosaic fathers**. *Am J Hum Genet* 1995, **56**:862-869.

41. Bondurand N, Kuhlbrodt K, Pingault V, Enderich J, Sajus M, Tommerup N, Warburg M, Hennekam RC, Read AP, Wegner M, Goossens M: **A molecular analysis of the yemenite deaf-blind hypopigmentation syndrome: SOX10 dysfunction causes different neurocristopathies**. *Hum Mol Genet* 1999, **8**:1785-1789.

42. Shahid M, Dhillon VS, Hussain Z, Masa JF, Aslam M, Raish M, Ahmad A, Khan NJ, Prasad S, Batra S, Pasha ST, Husain SA: **Analysis of the SRY gene in two sex-reversed XY sisters identifies two new novel point mutations in the high mobility group box domain**. *Fertil Steril* 2008, **90**:1199.

43. Schäffler A, Barth N, Winkler K, Zietz B, Rümmele P, Knüchel R, Schölmerich J, Palitzsch KD: **Identification of a new missense mutation (Gly95Glu) in a highly conserved codon within the high-mobility group box of the sex-determining region Y gene: report on a 46,XY female with gonadal dysgenesis and yolk-sac tumor**. *J Clin Endocrinol Metab* 2000, **85**:2287-2292.

44. Hawkins JR, Taylor A, Berta P, Levilliers J, Van der Auwera B, Goodfellow PN: **Mutational analysis of SRY: nonsense and missense mutations in XY sex reversal**. *Hum Genet* 1992, **88**:471-474.

45. Braun A, Kammerer S, Cleve H, Löhrs U, Schwarz HP, Kuhnle U: **True hermaphroditism in a 46,XY individual, caused by a postzygotic somatic point mutation in the male gonadal sex-determining locus (SRY): molecular genetics and histological findings in a sporadic case**. *Am J Hum Genet* 1993, **52**:578-585.

46. Jakubiczka S, Bettecken T, Stumm M, Neulen J, Wieacker P: **Another mutation within the HMG-box of the SRY gene associated with Swyer syndrome**. *Hum. Mutat* 1999, **13**:85.

47. Jäger RJ, Harley VR, Pfeiffer RA, Goodfellow PN, Scherer G: **A familial mutation in the testis-determining gene SRY shared by both sexes**. *Hum Genet* 1992, **90**:350-355.

48. Preiss S, Argentaro A, Clayton A, John A, Jans DA, Ogata T, Nagai T, Barroso I, Schafer AJ, Harley VR: **Compound effects of point mutations causing campomelic dysplasia/autosomal sex reversal upon SOX9 structure, nuclear transport, DNA binding, and transcriptional activation**. *J Biol Chem* 2001, **276**:27864-27872.

49. Zeng YT, Ren ZR, Zhang ML, Huang Y, Zeng FY, Huang SZ: **A new de novo mutation (A113T) in HMG box of the SRY gene leads to XY gonadal dysgenesis**. *J Med Genet* 1993, **30**:655-657.

50. Poulat F, Soullier S, Gozé C, Heitz F, Calas B, Berta P: **Description and functional implications of a novel mutation in the sex-determining gene SRY**. *Hum Mutat* 1994,**3**:200-204.

51. Jordan BK, Jain M, Natarajan S, Frasier SD, Vilain E: **Familial mutation in the testis-determining gene SRY shared by an XY female and her normal father**. *J Clin Endocrinol Metab* 2002, **87**:3428-3432.

52. Lundberg Y, Ritzen M, Harlin J : **Novel missense mutation (P131R) in the HMG box of SRY in XY sex reversal.** *Hum mutat* 1998 (Suppl 1):S328**.**
